# Effectiveness of Virtual Reality in Occupational Therapy for Post-Stroke Adults: A Systematic Review

**DOI:** 10.3390/jcm13164615

**Published:** 2024-08-07

**Authors:** Síbila Floriano Landim, Roberto López, Antonia Caris, Constanza Castro, Ramon D. Castillo, Daniela Avello, Braulio Henrique Magnani Branco, Pablo Valdés-Badilla, Florencia Carmine, Cristian Sandoval, Edgar Vásquez

**Affiliations:** 1 School of Occupational Therapy, Faculty of Psychology, Universidad de Talca, Talca 3465548, Chile; sibila.floriano@utalca.cl (S.F.L.); roberto.lopez@utalca.cl (R.L.); acaris20@alumnos.utalca.cl (A.C.); ccastro20@alumnos.utalca.cl (C.C.); 2 Graduate Program in Health Promotion, Cesumar University (UniCesumar), Maringá 87050-900, Brazil; braulio.branco@unicesumar.edu.br; 3 Centro de Investigación en Ciencias Cognitivas, Facultad de Psicología, Universidad de Talca, Talca 3465548, Chile; racastillo@utalca.cl; 4 Departamento de Terapia Ocupacional, Escuela de Ciencias de la Salud, Facultad de Medicina, Pontificia Universidad Católica de Chile, Santiago 7820436, Chile; daniela.avello@uc.cl; 5 Centro de Desarrollo de Tecnologías de Inclusión (CEDETI UC), Pontificia Universidad Católica de Chile, Santiago 7820436, Chile; 6 Department of Physical Activity Sciences, Faculty of Education Sciences, Universidad Católica del Maule, Talca 3530000, Chile; pvaldes@ucm.cl; 7 Sports Coach Career, School of Education, Universidad Viña del Mar, Viña del Mar 2520000, Chile; 8 Carrera de Medicina, Facultad de Medicina, Universidad de La Frontera, Temuco 4811230, Chile; f.carmine02@ufromail.cl; 9 Escuela de Tecnología Médica, Facultad de Salud, Universidad Santo Tomás, Los Carreras 753, Osorno 5310431, Chile; 10 Departamento de Medicina Interna, Facultad de Medicina, Universidad de La Frontera, Temuco 4811230, Chile; 11 Núcleo Científico y Tecnológico en Biorecursos (BIOREN), Universidad de La Frontera, Temuco 4811230, Chile

**Keywords:** cerebrovascular accident, neuronal plasticity, occupational therapy, rehabilitation, virtual reality

## Abstract

**Background:** In recent years, there has been a growing use of technological advancements to enhance the rehabilitation of individuals who have suffered from cerebrovascular accidents. Virtual reality rehabilitation programs enable patients to engage in a customized therapy program while interacting with a computer-generated environment. Therefore, our goal was to investigate the effectiveness of virtual reality in occupational therapy for people’s rehabilitation after a cerebrovascular accident. **Methods:** We systematically searched databases (Pubmed/Medline, Scopus, Web of Science, and Science Direct) for randomized controlled trials published within the last 10 years. Studies involving adult stroke survivors undergoing virtual reality-based interventions aimed at improving upper-extremity motor function were included. The quality assessment followed PRISMA guidelines, with the risk of bias assessed using the Cochrane tool (version 6.4) and methodological quality evaluated using GRADEpro. **Results:** We selected sixteen studies that met the main criteria for the implementation of virtual reality technology. The interventions described in the articles focused mainly on the upper extremities and their fine motor skills. **Conclusions:** When used in conventional treatments to improve people’s motor and cognitive functions after a cerebrovascular accident, virtual reality emerges as a beneficial tool. Additionally, virtual reality encourages adherence to the interventional process of rehabilitation through occupational therapy.

## 1. Introduction

Cardiovascular diseases, particularly cerebrovascular accidents (CVAs), stand as significant contributors to global mortality and disability, especially in developed nations [1]. Ischemic and hemorrhagic CVAs can cause a range of neurological problems, including paralysis, aphasia, and cognitive problems, because they cut off blood flow to the brain and damage neurons [2]. These conditions also lead to substantial physical impairments, including motor dysfunction and sensory deficits [2]. Globally, CVAs account for roughly 5,500,000 deaths per year and are a major contributor to long-term impairment [1]. Socioeconomic, demographic, and healthcare access factors further compound disparities in stroke incidence and outcomes, disproportionately affecting populations in low- and middle-income regions [2,3].

Effective rehabilitation post-CVA is crucial for optimizing recovery outcomes, with early intervention and occupational therapy emphasized to enhance functional independence and quality of life [4]. Rehabilitation interventions have shown promising results in improving physical and cognitive functions, particularly among individuals with severe CVA sequelae [5].

Recent technological advancements such as virtual reality (VR) have revolutionized CVA rehabilitation by providing immersive and customizable environments that facilitate neuroplasticity and aid in the recovery of motor and cognitive skills [6,7]. VR interventions have demonstrated notable enhancements in both motor and cognitive functions, as reported in various studies [8,9].

Perez-Marcos et al. [8] emphasize that VR can integrate crucial elements, such as motor–cognitive training and motivational aspects, thereby enhancing rehabilitation outcomes. However, challenges such as achieving optimal immersion and integrating haptic feedback remain areas of ongoing research and development [9].

In addition, adding games to VR-based methods has been demonstrated to significantly enhance patient engagement and adherence to therapy, which could lead to better functional outcomes [6]. Even though technology has come a long way, there is still not a lot of strong evidence about how well VR works in occupational therapy for people who have had a concussion. This underscores the need for further research to enhance rehabilitation strategies and ensure equitable access to novel treatments [10].

Other therapeutic approaches have also demonstrated efficacy in enhancing functionality and health-related quality of life in stroke survivors. For instance, Jaya Shanker Tedla et al. highlighted that constraint-induced movement therapy enhances upper- and lower-extremity motor activities and participation among stroke patients [11]. 

Hence, the objective of this systematic study is to synthesize and evaluate the present peer-reviewed literature to assess the effectiveness of VR in occupational therapy approaches for CVA rehabilitation. In particular, it aims to (i) describe the methods and outcomes of VR studies; (ii) look into how VR rehabilitation can help people get back to work after a CVA; and (iii) find out how well VR rehabilitation works for improving daily living skills after a CVA.

## 2. Methods

### 2.1. Protocol and Registration

This systematic review adhered to the PRISMA guidelines [12,13]. The PROSPERO database assigned the protocol registration number CRD42023472149. The report facilitates a comparison between the overall review and the protocol’s planned outcomes [14].

### 2.2. Eligibility

Eligibility requirements for the systematic review were original articles without any limitations on language or publication date, available up until May 2024. Excluded from consideration were books, conference abstracts, editorials, book chapters, letters to the editor, reviews, protocol records, trials, and case studies. To incorporate the studies into this systematic review, we utilized the PICOS framework (population, intervention, comparator, outcome, and study design), as shown in Table 1.

The following data were systematically extracted and analyzed from the selected studies: (i) title; (ii) author(s); (iii) year of publication; (iv) country of origin; (v) study design; (vi) main aim of the study; (vii) characteristics of the study population and number of participants; (viii) total duration of the intervention (weeks); (ix) weekly frequency and duration of each session; (x) key findings and outcomes reported; (xi) assessment of risk of bias; and (xii) level of evidence according to the Oxford Centre for Evidence-Based Medicine classification (Table 2).

### 2.3. Inclusion Criteria

Studies were included if they met the following criteria:

1. Study Design: RCTs with pre- and postintervention assessments. Only original research articles reporting primary data were included.
2. Population: Studies involving human participants diagnosed with CVA and sequelae, with a mean age of 18 years or older, and with no discrimination based on gender. The focus should be on adults with sequelae from CVA.
3. Interventions: Interventions utilizing VR specifically for recovery after CVA for a duration of six weeks. The interventions can be VR alone or VR combined with conventional rehabilitation.
4. Outcome Measures: Studies that report at least one evaluation related to quality of life, routine activities, or functional performance.
5. Comparison: Interventions should include a control group, with or without the use of VR.

### 2.4. Exclusion Criteria

Studies were excluded based on the following criteria:

1. Publication Type: Reviews, editorials, commentaries, conference abstracts, books, book chapters, letters to the editor, protocols, trials, and case studies.
2. Data Quality: Studies lacking detailed information on outcomes of interest, methodological clarity, or with insufficient initial data and/or lack of further monitoring.
3. Study Design: Non-randomized controlled trials, retrospective studies, cross-sectional studies, and prospective studies.
4. Animal Studies: Studies conducted on animal models.
5. Duplicate Publications: Duplicate studies reporting data from the same cohort or dataset.
6. Comparison: Absence of a control group in the study.

### 2.5. Database Search Process

The exploration process was conducted between August 2023 and May 2024 through four generic catalogs: Scopus, the core collection of Web of Science, Medline/PubMed, and Science Direct. MeSH terms from the National Library of Medicine of the United States of America used free language terms related to Leisure and Mild Cognitive Impairment. The following search string was used, for example, in Medline/PubMed: (((((“Virtual Reality”[Mesh] OR “Virtual Reality Exposure Therapy”[Mesh] OR “Exergaming”[Mesh]) OR “User-Computer Interface”[Mesh]) OR “Augmented Reality”[Mesh]) OR ((((((Virtual Reality Immersion Therapy)) OR (Virtual Reality Therapy)) OR (Virtual Systems)) OR (Immersive Virtual Reality)) OR (Non-Immersive Virtual Reality)) OR (Social Virtual Reality))) AND (((“Stroke”[Mesh]) OR “Cerebrovascular Disorders”[Mesh]) OR ((((Brain vascular disease) OR (Cerebrovascular Accident)) OR (Brain Vascular Accident)) OR (Cerebrovascular Stroke))) AND (((“Occupational Therapy”[Mesh]) OR ((Occupational Therapy Interventions) OR (Occupational Therapist)))).

The incorporated items and the inclusion and exclusion criteria were sent to two independent specialists to aid in identifying other pertinent studies. To evaluate the included manuscripts, the occupational therapy professionals had to meet two criteria: (i) hold a Ph.D. in Occupational Therapy, and (ii) have peer-reviewed publications on physical performance in diverse population clusters and/or in journals with an impact factor according to the Journal Citation Reports^®^. The research strategy was not disclosed to the experts to prevent any bias in their searches. After completing these steps, we conducted searches in the databases on 30 May 2024 to obtain pertinent errata or retractions associated with the studies included.

### 2.6. Study Selection and Data Collection Process

The studies were exported to Mendeley Reference Manager Version 2.116.1. Two authors (ACM and CCB) conducted independent searches, screened the titles, abstracts, and full texts, and removed duplicates. No disparities were found at this point. We then re-examined the full text of potentially acceptable articles, removing those that did not meet the selection criteria. Finally, two reviewers independently (EV and SFL) analyzed the entire data selection and extraction process in full.

### 2.7. Methodological Quality Assessment

The Centre for Evidence-Based Medicine, Oxford, uses a scale to assess the level of evidence in scientific articles. The Centre classifies articles into four grades of recommendation, ranging from A to the letter D, and further categorizes them into different levels of evidence, including 1a, 1b, 2a, 2b, 3a, 3b, 4, and 5. The hierarchical organization of this classification places level 1 as the best evidence and level 5 as the weakest or least solid evidence [31].

### 2.8. Data Synthesis

The following information was obtained and explored from the chosen studies: (i) title; (ii) author; (iii) year of publication; (iv) country of origin; (v) study design; (vi) main aim of the study; (vii) population and number of participants; (viii) total duration (weeks); (ix) weekly frequency and time per session; (x) main results of the studies; (xi) risk of bias; and (xii) level of evidence of the analyzed studies.

### 2.9. Risk of Bias Assessment

The method used to assess the risk of bias in the randomized controlled trials (RCTs) included in this review was ROB_2_ [32]. Two authors (AC and CC) independently completed the analysis, which was reviewed by another author (EV and SFL). We re-analyzed the original articles, identifying inconsistencies until we reached a consensus.

### 2.10. Measures for Meta-Analysis

The study protocol included meta-analyses. The full details are available on PROSPERO under the registry code CRD42023472149. However, the significant heterogeneity in the study designs, interventions, and outcomes measured precluded a robust meta-analysis.

### 2.11. Certainty of Evidence

We employed the GRADEpro scale (for the purposes of Grading of Recommendations, Assessment, Development, and Evaluation [32,33]) to evaluate the level of certainty of the evidence. We classified the articles as having high, moderate, low, or very low certainties of evidence. Due to the inclusion of studies with an RCT design, all examinations begun with a high level of certainty. Concerns regarding potential bias, uniformity, correctness, exactness, or the transparency of outcomes led to a downgrade. Two authors (AC and CC) conducted a separate evaluation of the research and resolved any differences through mutual agreement with two other authors (EV and SFL).

## 3. Results

### 3.1. Study Selection

**Figure 1:**
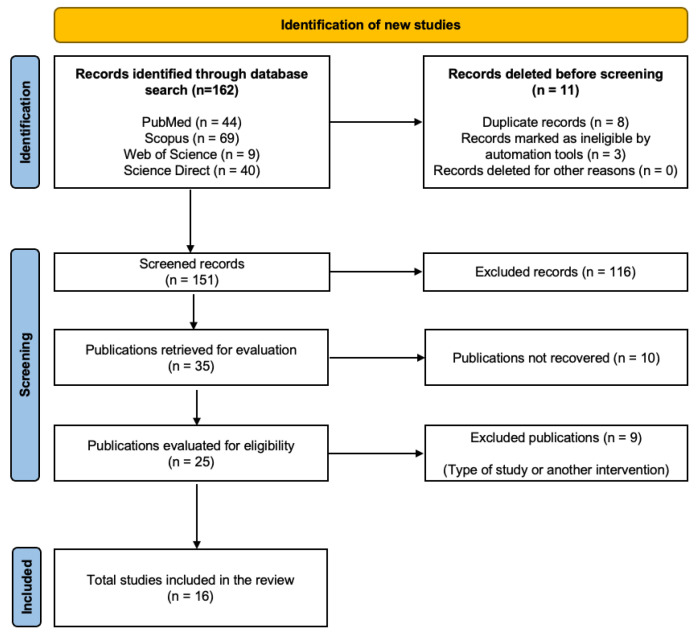
PRISMA flowchart.

The database search identified 162 articles, excluding 8 due to duplication and 3 due to automation. Out of the remaining 151 articles, we excluded 116 based on their title and abstract, and an additional 10 because we could not access the full text. After reviewing the full text of the 25 selected articles, we excluded 4 that were not RCTs and 5 for not addressing the anticipated intervention. Finally, we analyzed 16 articles [15,16,17,18,19,20,21,22,23,24,25,26,27,28,29,30]. Figure 1 outlines the study’s search process.

### 3.2. Methodological Quality Evaluation

The methodological quality of the articles included in this work is high because all sixteen are RCTs, reaching the highest level of evidence according to the Oxford scale, specifically level 1a. This design minimizes the risk of bias and provides a solid basis for reliably evaluating the impact of interventions.

### 3.3. Risk of Bias within Studies

**Figure 2:**
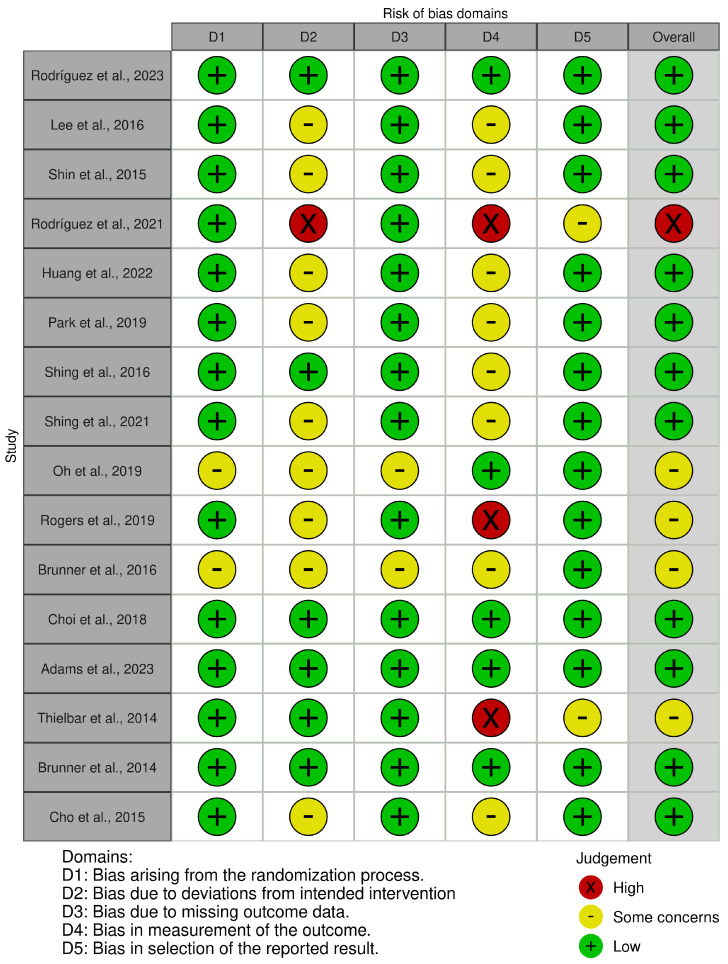
Risk of bias tool: traffic light chart [15,16,17,18,19,20,21,22,23,24,25,26,27,28,29,30].

**Figure 3:**
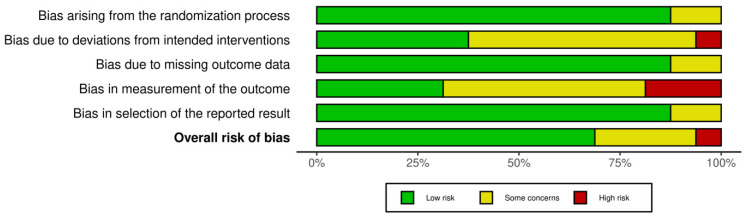
Risk of bias tool: summary chart by domain.

We found that sixteen studies had low potential for bias, and three had some concerns about bias. This suggests a low potential of bias in the research. Figure 2 and Figure 3 present a summary of the risk of bias.

### 3.4. Characteristics of the Studies

Table 3 lists the variables analyzed in the 16 selected studies. Three of these studies took place in Spain [15,18,22], seven in Korea [16,17,20,21,23,26,30], one in Taiwan [19], one in Australia [24], one in Norway [25], and three in the United States of America [27,28,29]. The systematic review included a total population of 555 participants. Of these, 322 were women (58.02%) and 233 were men (41.98%).

Table 3 categorizes each study based on its study design, the type of virtual technology used, its main aim, the intervention details, and its key findings. The studies involved diverse VR applications, such as specific VR, immersive VR, VR-based games, and VR combined with conventional therapy. They aimed to improve outcomes such as hand motor function, balance, postural stability, quality of life, and cognitive function in CVA survivors.

### 3.5. Sample Characteristics

The study participants’ age range was 18 to 94 years; 322 were women and 233 were men, with a diagnosis of CVA with predominant sequelae of motor impairment in the upper extremities and the ability to mainly follow instructions. All studies included conventional rehabilitation alone and the use of a VR device. Notably, 15 studies concentrated on enhancing the functionality of the upper limbs [15,17,18,19,20,21,22,23,24,25,26,27,28,29,30]. A single article focused solely on enhancing gait functionality and postural stability [16]. The authors presented structured sessions designed to significantly influence the participants. On average, all studies held sessions four days a week for a period of 4 to 8 weeks, with an intervention lasting 50 min. Among the most notable interventions across all studies were those related to fine motor work, range of motion, grasping, pinching, selective finger movements, strength training, and home-focused activities [15,16,17,18,19,20,21,22,23,24,25,26,27,28,29,30].

The most frequently used assessment instruments in the analyzed articles were the Fugl-Meyer/FMA-UE [34], FIM [35], ARAT [36], and CVA Impact Scale 3.0 [37].

The Hamilton Depression Scale (HDS) [38], SF-36 [39], Wolf’s Motor Function Test [40], EQ-5D-5L instrument, modified Ashworth Scale [41], and EuroQoL visual analog scale (EQ-VAS) [42], among others, were some of the additional scales and instruments used to evaluate various areas of motor functioning, health-related quality of life, and depression. These evaluation scales and instruments enable the assessment of areas affected by a CVA and occupational performance, providing pertinent information to understand an individual’s current state. This information enables interventions to be adapted in the most equitable manner possible.

### 3.6. Dosage and Interventions Performed

Throughout the interventions, VR was integrated into all occupational therapy programs. These programs encompassed a wide range of recreational activities and exercises designed to improve both the cognitive and physical health of participants. The activities, meticulously planned and carried out by occupational therapists, actively included virtual reality, thus promoting cognitive, emotional, and physiological benefits throughout the studies.

### 3.7. Data Collection Instruments

The RTC analysis focused on measuring VR’s influence on people with stroke sequelae. Four studies evaluated general cognitive function using the Mini Mental State Examination scale (MiniMental) [43]. Eight studies used the SF-36 scale to evaluate quality of life [39]. Seven studies used the Fugl-Meyer scale to evaluate manual function and daily activities [34], three used Barthel, and eight used FIM.

### 3.8. Adverse Effects and Adherence

The studies included in the systematic review did not report adverse effects and demonstrated high adherence rates among participants. Adherence percentages varied between 78% and 92% across the studies.

### 3.9. Certainty of Evidence

The certainty of the evidence allows us to make recommendations about virtual reality as a good intervention tool in occupational therapy for people with stroke sequelae (Table 4).

## 4. Discussion

Our systematic review aimed to assess VR’s effectiveness in occupational therapy for post-CVA individuals. The findings consistently highlight VR as a potent adjunct to conventional rehabilitation, particularly in enhancing UEMF, ADL performance, and overall quality of life [15,16,18,21,23,26,30]. This result demonstrates the importance of considering VR as an intervention strategy that helps enhance the rehabilitation of people with stroke sequelae and opens the question of whether it can serve as a therapeutic tool for other pathologies of acquired brain damage. VR is a novel, interesting, and motivating complement to the rehabilitation process, which is key to improving sensory, motor, and cognitive functions. The improvement of these functions has an objective impact on stroke patients’ independence and quality of life. This intervention tool is becoming increasingly affordable and accessible to a larger number of health centers and universities. The studies conducted by Shin et al. [21] and Lee et al. [16] show significant improvements in upper-limb distal motor function and postural control. Patients in these studies got much better at controlling their posture and the distal motor function of their upper limbs. This was because virtual reality helped them to perform exercises that improve neuroplastic changes, muscle strength, coordination, and proprioception [1]. These physiological adaptations are crucial to restoring functional abilities after stroke, especially in older adults who face greater challenges in motor recovery [2]. Furthermore, Cho et al. [30] highlighted VR’s cognitive benefits in dual-task conditions related to walking function, suggesting broader applications beyond motor rehabilitation. VR engages multiple sensory modalities simultaneously, enhancing neural activation and cognitive processing [3].

### 4.1. Data Collection Instruments Used

A variety of assessment tools were used, including the Functional Movement Assessment of the Upper Limbs (FMA-UE), Grip and Reach Ability Test (ARAT), Functional Independence Measure (FIM), and Stroke Impact Scale (SIS). These tools comprehensively assessed motor function, performance in activities of daily living, quality of life, and psychological well-being across the studies [4].

### 4.2. Types of Interventions

The VR interventions varied in terms of immersion and specific objectives, and included UUEE rehab, balance training, posture control, gait improvement, and cognitive tasks. These immersive interventions optimize motor learning with repetition, intensity, and task specificity, improving neuroplasticity, recovery, and functional independence [5].

### 4.3. Outcomes Reported and GRADE Assessment

Using the GRADE framework, the RCTs consistently showed moderate-to-high-certainty evidence that VR can help improve motor function in the upper limbs, ADL performance, and quality of life after a CVA. Effect sizes indicated clinically significant improvements (ES = 0.5–0.8), highlighting the robustness of VR in clinical settings [6].

### 4.4. Strengths and Future Directions

Despite promising results, there were some limitations at play, including studies with short follow-up periods and populations with diverse sequelae. Future research should focus on longitudinal evaluations to capture sustained benefits. The integration of VR with emerging technologies such as AI, robotics, home automation, and functional exoskeletons could further enhance and improve rehab outcomes, as well as personalize interventions according to the needs of CVA patients.

### 4.5. Limitations

Despite the promising findings, our systematic review has several methodological limitations that should be considered when interpreting the results. Firstly, there was heterogeneity in the study designs and populations evaluated, which may have introduced biases and made direct comparisons challenging. The included studies exhibited significant variability in their methodologies and participant demographics. The studies originated from diverse geographical locations, such as Spain, Korea, Taiwan, Australia, Norway, and the United States, with differing healthcare systems and rehabilitation practices. This variability in study designs and populations may impact the generalizability of the findings across different clinical settings and patient groups. For instance, cultural and regional differences could influence patient responses to VR interventions, affecting the reproducibility of results in other contexts.

The total population across the 16 studies included 555 participants, with varying sample sizes per study. While most participants were middle-aged, the age range extended from 18 to 94 years. Moreover, the distribution between men (41.98%) and women (58.02%) was uneven across studies, potentially influencing outcomes given the known differences in stroke incidence and recovery between genders. The small sample sizes in some studies may have limited statistical power and affected the reliability of the observed effects, particularly when subgroup analyses were considered.

Although all studies employed RCT designs, there were differences in the specific VR technologies used, the duration and intensity of interventions, and the types of outcome measures employed. These variations complicate direct comparisons and highlight the challenge of synthesizing findings across studies. Moreover, the use of different assessment tools for motor function, quality of life, and other outcomes introduces additional complexity when pooling results for meta-analysis or drawing conclusive interpretations.

Additionally, we assessed the methodological quality and risk of bias of some included studies as moderate or high using risk-of-bias assessment tools. This variability in study quality could potentially affect the validity and reliability of the reported outcomes. In fact, the choice of outcome measures varied widely among studies, including assessments like the Fugl-Meyer Assessment, ARAT, and SF-36. Because of these problems, more research needs to be completed to find ways to fix any biases and make sure that study designs are consistent so that the results and clinical uses of VR interventions in post-concussion rehabilitation are stronger.

## 5. Conclusions

Virtual reality emerges as a therapeutic tool in occupational therapy aimed at improving functional outcomes and quality of life among individuals recovering from CVAs. Despite the need for meta-analyses to fully verify its effectiveness, clinicians can optimize neurorehabilitation strategies, enhance motor learning, and facilitate recovery across different age groups by leveraging VR’s immersive capabilities and targeted interventions. Future research and clinical practice should continue to explore innovative approaches and address challenges to maximize the potential of VR in enhancing rehabilitation outcomes and promoting long-term independence and well-being.

## Figures and Tables

**Table 1:** The systematic review employed specific selection criteria.

Criteria	Inclusion	Exclusion
Population	Adults with sequelae after a CVA considered as participants, with a mean age of 18 years or older, and with no discrimination based on gender	Adults with pathologies other than stroke or people younger than 18 years old
Intervention	Interventions used VR for recovery for six weeks after CVA	Method did not contain VR interventions for recovery after a stroke
Comparison	Interventions included a CG, with or without the use of VR	Absence of CG
Outcomes	At least one evaluation of quality of life, routine activities, or functional performance	Insufficient initial data and/or further monitoring
Study design	RCT with pre- and post-assessment	Non-RCT, retrospective, cross-sectional and prospective studies

CG: control group; CVA: cerebrovascular accidents; RCT: randomized controlled trial; VR: virtual reality.

**Table 2:** Level of evidence according to Oxford classification.

References	Name of Study	Type of Study	Level of Evidence According to Oxford Classification
[15]	Can specific virtual reality combined with conventional rehabilitation improve poststroke hand motor function? A randomized clinical trial	RCT	1a
[16]	Canoe game-based virtual reality training to improve trunk postural stability, balance, and upper limb motor function in subacute stroke patients: A randomized controlled pilot study	RCT	1a
[17]	Effects of game-based virtual reality on health-related quality of life in chronic stroke patients: A randomized, controlled study	RCT	1a
[18]	Effects of Specific Virtual Reality-Based Therapy for the Rehabilitation of the Upper Limb Motor Function Post-Ictus: Randomized Controlled Trial	RCT	1a
[19]	Effects of virtual reality-based motor control training on inflammation, oxidative stress, neuroplasticity and upper limb motor function in patients with chronic stroke: a randomized controlled trial	RCT	1a
[20]	Effects of virtual reality-based planar motion exercises on upper extremity function, range of motion, and health-related quality of life: A multicenter, single-blinded, randomized, controlled pilot study	RCT	1a
[21]	Effects of virtual reality-based rehabilitation on distal upper extremity function and health-related quality of life: A single-blinded, randomized controlled trial	RCT	1a
[22]	Effects of virtual reality-based therapy on quality of life of patients with subacute stroke: A three-month follow-up randomized controlled trial	RCT	1a
[23]	Efficacy of Virtual Reality Combined with Real Instrument Training for Patients with Stroke: A Randomized Controlled Trial	RCT	1a
[24]	Elements virtual rehabilitation improves motor, cognitive, and functional outcomes in adult stroke: Evidence from a randomized controlled	RCT	1a
[25]	Is upper limb virtual reality training more intensive than conventional training for patients in the subacute phase after stroke? An analysis of treatment intensity and content	RCT	1a
[26]	Mobile game-based virtual reality program for upper extremity stroke rehabilitation	RCT	1a
[27]	Telehealth-Guided Virtual Reality for Recovery of Upper Extremity Function Following Stroke	RCT	1a
[28]	Training finger individuation with a mechatronic-virtual reality system leads to improved fine motor control post-stroke	RCT	1a
[29]	Virtual reality training for upper extremity in subacute stroke (VIRTUES): Study protocol for a randomized controlled multicenter trial	RCT	1a
[30]	Virtual reality training with cognitive load improves walking function in chronic stroke patients	RCT	1a

RCT: randomized controlled trial.

**Table 3:** Summary of virtual reality studies in post-CVA patients.

References	Study Design	Population Settings	Type of Virtual Technology Used	Main Aim	Intervention	Main Findings
[15]	RCT	Total: 43, EG: 23, CG: 20, Middle age: 60, Women: 30, Men: 13	Specific virtual reality	To improve hand motor function post-CVA using CT and VR	15 OT sessions over 3 weeks, 150 min each	Improved motor function using SVR compared to CT
[16]	RCT	Total: 10, EG: 5, CG: 5, Middle age: 62, Women: 7, Men: 3	Canoe game- based VR	To improve trunk stability and UUEE function post-CVA using VR	12 OT sessions over 4 weeks, 30 min/day	Positive impact on trunk stability and motor function using VR
[17]	RCT	Total: 32, EG: 16, CG: 16, Middle age: 57, Women: 20, Men: 12	Rehab Master system VR	To improve HRQoL, depression, and UUEE function using VR and OT	20 OT sessions over 4 weeks, 30 min/day	Enhanced quality of life and UUEE function using VR + OT compared to OT
[18]	RCT	Total: 43, EG: 23, CG: 20, Middle age: 59, Women: 30, Men: 13	Virtual reality	To improve UUEE motor function post-CVA using physical therapy + OT + SVR	15 OT sessions over 3 weeks, 150 min each	Significant improvement in motor function using physical therapy + OT + SVR compared to physical therapy + OT
[19]	RCT	Total: 30, EG: 15, CG: 15, Middle age: 59, Women: 20, Men: 10	Immersive VR	To enhance motor control and reduce inflammation post-CVA using VR	16 OT sessions, 60 min/day, 2–3 days/week, in addition to attending regular occupational therapy	Improved motor control and reduced inflammation
[20]	RCT	Total: 25, EG: 12, CG: 13, Middle age: 65, Women: 20, Men: 5	Planar motion VR	To evaluate feasibility for UUEE intervention using VR	20 OT sessions over 4 weeks, 30 min/day	Promising feasibility for UUEE rehabilitation using VR + OT compared to OT
[21]	RCT	Total: 33, EG: 20, CG: 13, Middle age: 60, Women: 20, Men: 13	VR-based OT	To improve distal UUEE function and HRQoL using VR + OT in CVA survivors	20 OT sessions over 4 weeks, 30 min/day	Better distal function and quality of life using VR + OT compared to OT
[22]	RCT	Total: 43, EG: 23, CG: 20, Middle age: 63, Women: 31, Men: 12	Virtual reality	To enhance post-CVA HRQoL using CT + VR	15 OT sessions over 3 weeks, 150 min each	Improved health-related quality of life using CT + VR
[23]	RCT	Total: 31, EG: 17, CG: 14, Middle age: 59, Women: 11, Men: 10	Joystim VR	To improve cognitive and UUEE function post-CVA using VR	18 OT sessions over 6 weeks, 30 min/day	Positive impact on cognitive and motor functions using VR
[24]	RCT	Total: 21, EG: 10, CG: 11, Middle age: 63, Women: 5, Men: 16	Elements VR	Rehabilitate motor and cognitive functions using VR	12 OT sessions over 4 weeks, 30–40 min each	Effective for motor and cognitive rehabilitation using VR
[25]	RCT	Total: 50, EG: 25, CG: 25, Middle age: 62, Women: 31, Men: 29	YouGrabber system VR	To compare VR training intensity with CT	16 OT sessions over 4 weeks, 45–60 min/day	Comparable intensity with CT
[26]	RCT	Total: 24, EG: 12, CG: 12, Middle age: 61, Women: 12, Men: 12	Mobile game VR	To develop mobile game-based VR program	10 OT sessions over 2 weeks	Promising for upper-extremity rehabilitation using a mobile game-based VRprogram
[27]	RCT	Total: 18, EG: 9, CG: 9, Middle age: 67, Women: 17, Men: 11	GRASP system VR	To evaluate home-based VR program for UUEE recovery post-CVA	16 OT sessions over 8 weeks, 4 sessions/week	Home-based VR program resulted in effective UUEE recovery post-CVA
[28]	RCT	Total: 14, EG: 7, CG: 7, Middle age: 60, Women: 8, Men: 6	Mechatronic VR system	To improve fine motor control post-CVA using a mobile game-based VR program	18 OT sessions over 6 weeks, 60 min/day	Improved fine motor control post-CVA
[29]	RCT	Total: 106, EG: 53, CG: 53, Middle age: 62, Women: 50, Men: 56	Virtual reality	To compare VR + CT vs. CT to improve arm motor function after CVA	20 OT sessions over 4 weeks, 45–60 min/day	Improve arm motor function post-CVA using VR + CT compared to CT
[30]	RCT	Total: 22, EG: 11, CG: 11, Middle age: 60, Women: 10, Men: 12	Virtual reality	Compare VR vs. conventional physical therapy and OT	30 OT sessions over 6 weeks, 30 min/day	Significant improvement in walking function using VR

CG: control group; CT: conventional therapy; CVA: stroke; EG: experimental group; GRASP: Virtual Reality-Based Home Exercise Program Virtual Reality (Graded Repetitive Arm Supplementary Program); HRQoL: health-related quality of life; OT: occupational therapy; RCT: Ensayo Clínico Aleatorizado (randomized controlled trial); SVR: specific virtual reality; UUEE: upper-extremity motor function; VR: virtual reality.

**Table 4:** Methodological quality assessment using GRADEpro tool (https://www.gradeworkinggroup.org/; accessed on 30 May 2024).

Certainty of Evidence							No. of Patients		Effect	Certainty	Importance
References	Study Design	Risk Assessment	Inconsistency	Indirect Evidence	Vagueness	Other Considerations	[Conventional Therapy plus Virtual Reality]	[Conventional Therapy]	Relative (95% CI)		
Rodríguez et al. [15]	RCT	Not serious	Not serious	Not serious	Not serious	None	23/46 (50%)	23/46 (50%)	Not estimable	++++ High	IMPORTANT
Lee et al. [16]	RCT	Not serious	Not serious	Not serious	Not serious	None	5/10 (50%)	5/10 (50%)	Not estimable	++++ High	IMPORTANT
Shin et al. [17]	RCT	Not serious	Not serious	Not serious	Not serious	None	8/16 (50%)	8/16 (50%)	Not estimable	++++ High	IMPORTANT
Rodríguez et al. [18]	RCT	Serious ^a^	Serious	Not serious	Not serious	Publication bias seriously suspected ^a^	23/43 (53.5%)	20/43 (46.5%)	Not estimable	+ Very low	IMPORTANT
Huang et al. [19]	RCT	Not serious	Not serious	Not serious	Not serious	None	15/30 (50%)	15/30 (50%)	Not estimable	++++ High	IMPORTANT
Park et al. [20]	RCT	Not serious	Not serious	Not serious	Not serious	None	12/25 (48%)	13/25 (52%)	Not estimable	++++ High	IMPORTANT
Shin et al. [21]	RCT	Not serious	Not serious	Not serious	Not serious	None	23/46 (50%)	23/46 (50%)	Not estimable	++++ High	IMPORTANT
Rodríguez et al. [22]	RCT	Not serious	Not serious	Not serious	Not serious	None	23/46 (50%)	23/46 (50%)	Not estimable	++++ High	IMPORTANT
Oh et al. [23]	RCT	Not serious	Not serious	Not serious	Not serious	None	17/31 (54.8%)	14/31 (45.2%)	Not estimable	++++ High	IMPORTANT
Rogers et al. [24]	RCT	Very serious ^b^	Serious ^b^	Serious ^b^	Not serious	None	10/21 (47.6%)	11/21 (52.4%)	Not estimable	+ Very low	IMPORTANT
Brunner et al. [25]	RCT	Not serious	Not serious	Not serious	Not serious	None	25/50 (50%)	25/50 (50%)	Not estimable	++++ High	IMPORTANT
Choi et al. [26]	RCT	Not serious	Not serious	Not serious	Not serious	None	12/24 (50%)	12/24 (50%)	Not estimable	++++ High	IMPORTANT
Adams et al. [27]	RCT	Not serious	Not serious	Not serious	Not serious	None	9/18 (50%)	9/18 (50%)	Not estimable	++++ High	IMPORTANT
Thielbar et al. [28]	RCT	Serious ^c^	Not serious	Serious	Not serious	None	8/16 (50%)	8/16 (50%)	Not estimable	++ Low	IMPORTANT
Brunner et al. [29]	RCT	Not serious	Not serious	Not serious	Not serious	None	60/120 (50%)	60/120 (50%)	Not estimable	++++ High	IMPORTANT
Cho et al. [30]	RCT	Not serious	Not serious	Not serious	Not serious	None	12/24 (50%)	12/24 (50%)	Not estimable	++++ High	IMPORTANT

**Authors:** Antonia Caris and Constanza Castro. **Question:** [Conventional therapy plus virtual reality] compared with [conventional therapy] for [cerebrovascular accident]. ^a^ There is a risk of bias because the participants may have been aware of the intervention, and the trial’s context may have influenced the study’s implementation and results. This is because the COVID-19 context made it challenging to follow up with the participants and assess their progress six months after the combined treatment was complete. ^b^ Pilot study that was not registered; it is also highlighted that the sample was very limited, and lastly, the evaluators were not blinded. ^c^ Only one therapist who was part of the research was blinded, so there is a possibility that the process may have been influenced in relation to the results. 9-HPT: 9-Hole Peg Test; ARAT: Action Arm Research Test; BBT: Box and Block Test; CI: confidence interval; CVA: cerebrovascular accident; EQ-5D-5L: EuroQoL-5 Dimensions Instrument; EQ-VAS: EuroQoL Visual Analog Scale; FIM: Functional Independence Measure; FMA-UE: Fugl-Meyer Assessment—Upper Extremity; FMA-UE/FMA: Fugl-Meyer Assessment—Upper Extremity/Hand Subcomponent; GMLT: GMLT: Groton Maze Learning Task from the CogState computerized assessment battery; GS: grip strength; JTHFT: Jebsen–Taylor Hand Function Test; K-MMSE: Korean Mini-Mental State Examination; K-MoCA: Korean Montreal Cognitive Assessment; LPS: lateral pinch strength; MoCA: Montreal Cognitive Assessment; NFI: Neurobehavioural Functioning Inventory; PPS: palmar pinch strength; RCT: randomized controlled trial; SST: Set Shift Task from the CogState computerized assessment battery; UUEE: upper extremities; VR: virtual reality training; VR: virtual reality.

## Data Availability

The authors confirm that the data supporting the findings of this study are available within the article.
